# Application of RR-XGBoost combined model in data calibration of micro air quality detector

**DOI:** 10.1038/s41598-021-95027-1

**Published:** 2021-08-02

**Authors:** Bing Liu, Xianghua Tan, Yueqiang Jin, Wangwang Yu, Chaoyang Li

**Affiliations:** 1 Public Foundational Courses Department, Nanjing Vocational University of Industry Technology, Nanjing, 210023 China; 2 School of Mechanical Engineering, Nanjing Vocational University of Industry Technology, Nanjing, 210023 China; 3 College of Management, Henan University of Technology, Zhengzhou, 450001 China

**Keywords:** Atmospheric science, Climate change, Climate sciences, Environmental sciences, Environmental impact

## Abstract

Grid monitoring is the current development direction of atmospheric monitoring. The micro air quality detector is of great help to the grid monitoring of the atmosphere, so higher requirements are put forward for the accuracy of the micro air quality detector. This paper presents a model to calibrate the measurement data of the micro air quality detector using the monitoring data of the air quality monitoring station. The concentration of six types of air pollutants is the research object of this study to establish a calibration model for the measurement data of the micro air quality detector. The first step is to use correlation analysis to find out the main factors affecting the concentration of the six types of pollutants. The second step uses Ridge Regression (RR) to select variables, find out the factors that have significant effects on the concentration of pollutants, and give the quantitative relationship between these factors and the pollutants. Finally, the predicted value of the ridge regression model and the measurement data of the micro air quality detector are used as input variables, and the Extreme Gradient Boosting (XGBoost) algorithm is used to give the final pollutant concentration prediction model. We named the combined model of ridge regression and XGBoost algorithm RR-XGBoost model. Relative Mean Absolute Percent Error (MAPE), Mean Absolute Error (MAE), goodness of fit (*R*^2^), and Root Mean Square Error (RMSE) were used to evaluate the prediction accuracy of the RR-XGBoost model. The results show that the model is superior to some commonly used pollutant prediction methods such as random forest, support vector machine, and multilayer perceptron neural network in the evaluation of various indicators. The model not only has a good prediction effect on the training set but also on the test set, indicating that the model has good generalization ability. Using the RR-XGBoost model to calibrate the data of the micro air quality detector can make up for the shortcomings of the data monitoring accuracy of the micro air quality detector. The model plays an active role in the deployment of micro air quality detectors and grid monitoring of the atmosphere.

## Introduction

Air pollutants are composed of a mixture of gaseous, volatile, semi-volatile and particulate matter, and their composition is relatively complex. The concentration of air pollutants is affected by many factors, including meteorological conditions, different time periods, industrial activities, and traffic intensity. In recent years, researchers have paid more and more attention to the relationship between air pollution and various human diseases, especially lung disease and cardiovascular disease^1,2^. According to statistics, outdoor air pollution causes more than 3 million premature deaths worldwide every year. If outdoor air pollution emissions remain unchanged, the premature death caused by outdoor air pollution may double by 2050, and it is estimated that 6.6 million premature deaths will be caused each year^3,4^. Therefore, the monitoring of air pollutant concentration has received more and more attention from relevant departments.

### Air quality monitoring platform

In response to the problem of pollutant concentration monitoring, some countries have set up air quality monitoring stations (national control points) in their key areas. The national control point is excellent in the accuracy of pollutant concentration monitoring, but its maintenance and construction costs are high, resulting in a small number of settings, and the pollutant concentration in most areas cannot be monitored. In addition, the release of national control point data is lagging, making it difficult for relevant departments to timely control pollution sources through pollutant data.

In order to overcome the deficiencies of national control points in air quality monitoring, micro air quality detectors (self-built points) are often used to monitor the concentration of pollutants. The electrochemical sensor module is an important part of the micro air quality detector. When there is a detectable gas, the gas and the electrochemical sensor produce oxidation or reduction reactions, and a weak current is generated, which is output on the electrode. The output current has a linear relationship with the gas concentration. Detecting the output current of the electrode can calculate the concentration value of the gas.

The micro air quality detector is easy to install, and its cost is low, which is conducive to grid deployment. In addition, the self-built point indicator is easy to read, which is conducive to real-time monitoring of air quality^5–7^. Since the electrochemical sensor used in the micro air quality detector is very sensitive to temperature and humidity, when the environment changes greatly, the measurement accuracy will be affected to a certain extent. In addition, the zero point and range shift of the electrochemical sensor during use for a period of time will cause errors in the measurement concentration. Therefore, compared with the monitoring data of national control points, the accuracy of the data measured by self-built points needs to be improved.

### Introduction to pollutant concentration prediction model

Air pollutants mainly include O_3_, PM2.5, PM10, CO, NO_2_, and SO_2_ (“two dust and four gases”). Many air quality assessment indicators take the concentration of "two dust and four gases" as an important basis. At present, a variety of algorithm models have been used by scholars at home and abroad to predict the concentration of pollutants in the atmosphere, and relatively good results have been achieved. These model algorithms mainly include time series models, chemical transmission models, machine learning models, etc.

The time series models used to predict air quality include: Moving Average (MA) model, Autoregressive (AR) model, Autoregressive Moving Average (ARMA) model, Autoregressive Integral Moving Average (ARIMA) model, fuzzy time series model, etc. Jian et al. used the ARIMA model to successfully predict the concentration of PM1.0 in the street area^8^. Koo et al. used ARIMA and Singh fuzzy time series model and other models to predict the air pollution index of Kuala Lumpur, Malaysia in 2017. After comparison, it is found that the Singh fuzzy time series model is the most accurate and effective forecasting model^9^.

The chemical transport model is based on scientific theories and assumptions. It uses numerical methods combined with meteorological principles to simulate and describe processes such as the transmission, diffusion, and chemical reactions of pollutants in the atmosphere. The chemical transmission model obtains the pollutant concentration distribution by inputting the source emission, topography, meteorological data, and operation mode of the study area^10–12^. Because the pollutant formation and transmission process is very complicated, the calculation complexity of the chemical transmission model is relatively high, and the model accuracy is not high.

Since the linear regression model is convenient to explain the quantitative relationship between pollutants and other variables of the model, the multivariate linear regression model is still a commonly used pollutant concentration prediction model^13–15^. The artificial neural network model combined with an effective training algorithm can detect the complex and potentially non-linear relationship between the predictor variable and the response variable, and this model has become the current mainstream^13,16–18^. In addition, prediction methods such as Markov chain^19–21^, support vector machine^22–24^, and random forest^25–27^are also commonly used to predict the concentration of air pollutants. Because Extreme Gradient Boosting (XGBoost) has excellent computing efficiency and prediction accuracy, it has also been widely used in the prediction of air pollutant concentration in recent years. Zhai et al. used LASSO, Adaboost, XGBoost and other algorithms to integrate with support vector regression, and successfully predicted the daily average concentration of PM2.5 in Beijing, China^28^. Joharestani et al. used Random Forest, XGBoost, and Deep Learning to predict PM2.5 concentration, and the results showed that the model performance obtained by using the XGBoost algorithm was the best^29^.

## Material and methods

### Data source and preprocessing

The insufficient measurement accuracy of the micro air quality detector is an important factor affecting its promotion. In order to establish the measurement data correction model of the micro air quality detector, this study collected two sets of data. The first set of data comes from an air quality monitoring station in Nanjing, which is considered accurate data in this study. It contains 4200 samples, which records the hourly concentration of six pollutants from November 14, 2018 to June 11, 2019. The second set of data is provided by the micro air quality detector and the location of the micro air quality detector is juxtaposed with the air quality monitoring station. Electrochemical sensors are used in the monitoring equipment of the micro air quality detector. 234,717 samples are included in the second set of data, and the time interval between each sample does not exceed 5 min. The micro air quality detector not only provides the concentration of six pollutants, but also provides five meteorological parameters including wind speed, pressure, precipitation, temperature and humidity. Due to the insufficient accuracy of the measurement data of the micro air quality detector, it is necessary to establish a pollutant concentration correction model to correct the measurement data.

Before constructing the data correction model of the micro air quality detector, the original data should be preprocessed. First, remove the outliers in the measurement data of the self-built points. In this paper, data whose measured value is greater than 3 times the average value of the left and right adjacent data or less than 1/3 times the average value of the left and right adjacent data are regarded as the outlier. Then calculate the hourly average of the self-built point measurement data, in order to correspond with the national control point measurement data. For the data whose self-built point cannot correspond to the national control point, this article directly deletes them. After preprocessing, a total of 4135 samples were obtained^13,24^. Table 1 describes the variables contained in the samples.

**Table 1:** Descriptive statistics of pollutant concentrations and meteorological parameters measured by national control points and self-built points.

Input variable	Ranges	Mean	Standard deviation	Skewness	Kurtosis
PM2.5/(μg/m^3^)	1–216.883	64.127	37.328	0.988	0.701
PM10/(μg/m^3^)	2–443.25	102.391	65.267	1.476	2.862
CO/(μg/m^3^)	0.05–3.895	0.863	0.452	1.463	3.136
NO_2_/(μg/m^3^)	0.947–157.136	45.209	28.403	0.653	− 0.259
SO_2_/(μg/m^3^)	1–651.3	19.397	18.723	12.781	342.11
O_3_/(μg/m^3^)	0.579–259	61.586	40.941	1.091	2.035
Wind speed/(m/s)	0.133–2.387	0.7	0.346	0.862	0.748
Pressure/(Pa)	996.871–1039.8	1018.8	8.889	− 0.093	− 0.599
Precipitation/(mm/m^2^)	0–312.1	132.084	87.004	0.245	− 0.728
Temperature/(℃)	− 3.882 to 37.944	11.882	8.603	0.625	− 0.399
Humidity/(rh%)	10.667–100	68.903	21.931	− 0.487	− 0.756

### Data exploratory analysis

Because the research methods of the six types of pollutants concentration are similar, this paper selects O_3_ concentration as the main research object. The ozone in the atmosphere is divided into tropospheric near-ground ozone and stratospheric ozone. What is harmful to the environment and human health is near-surface ozone in the troposphere, also known as bad ozone. If humans are exposed to bad ozone for a long time, it will cause damage to the respiratory system and immune system.

**Figure 1:**
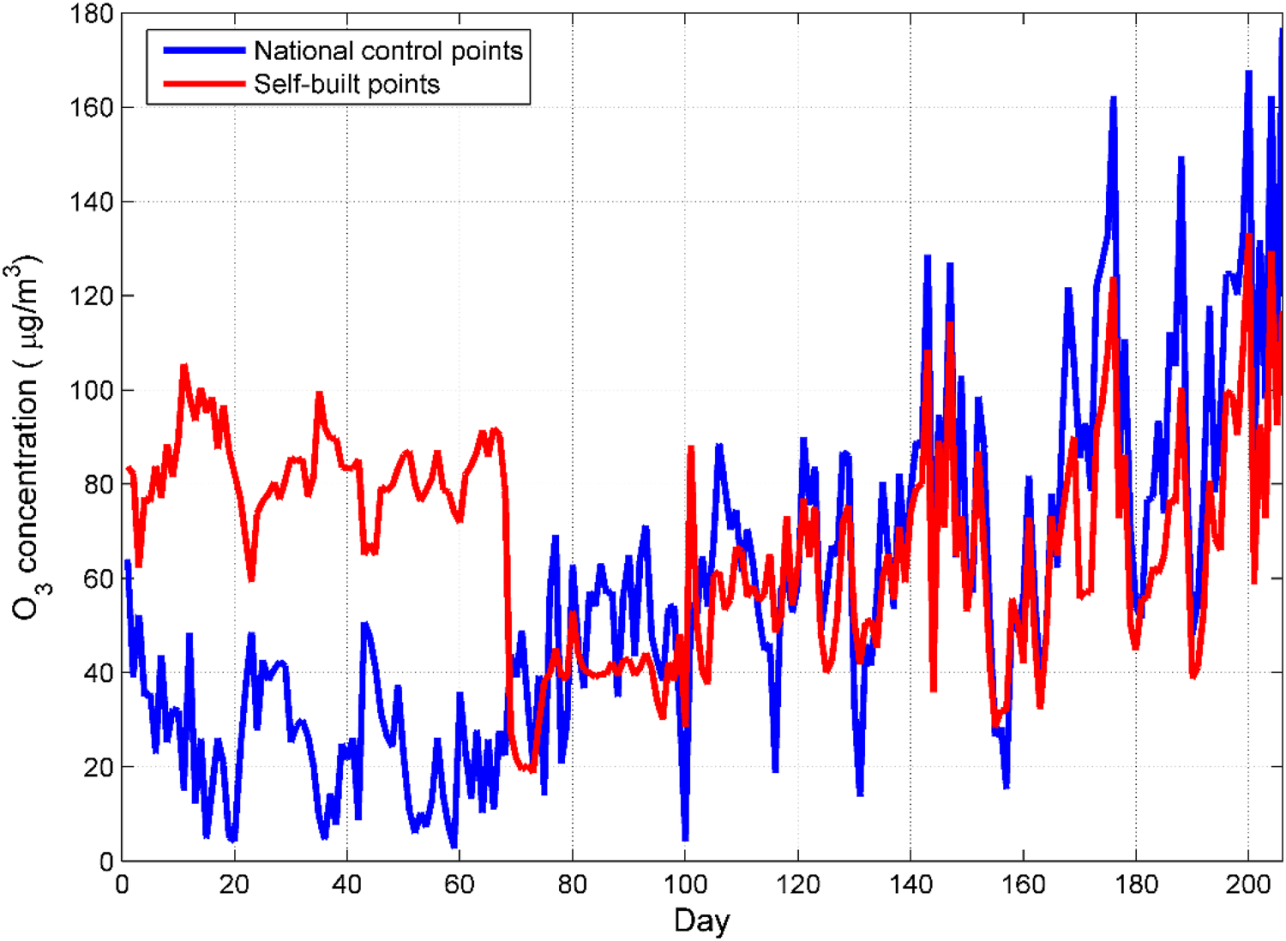
Comparison of daily average O_3_ concentration data between national control points and self-built points. Figures are generated using Matlab (Version R2016a, https://www.mat- hworks.com/) [Software].

Before establishing the data correction model of the micro air quality detector, it is necessary to perform descriptive statistics on the data in order to grasp the overall trend of the pollutant concentration in the air and the measurement error of the micro air quality detector^15,30^. Because too much sample data is not conducive to visually analyzing the change trend of air pollutant concentration and the measurement error of the micro air quality detector, we calculated the daily average of the O_3_ concentration. After the data were averaged, a total of 206 sets of data were obtained^31^. It can be seen from Fig. 1 that the O_3_ concentration of the self-built point and the national control point are in good agreement in the later period, but there is a certain deviation in the previous period. The low temperature and huge changes in humidity in autumn and winter interfere with the electrochemical sensor, which leads to deviations in the measurement data of the micro air quality detector. In addition, the obvious difference in O_3_ concentration in different time periods can also be seen from Fig. 1. In order to visually reflect the difference of O_3_ concentration in different time periods, this paper draws a box plot of O3 concentration changes with months.

**Figure 2:**
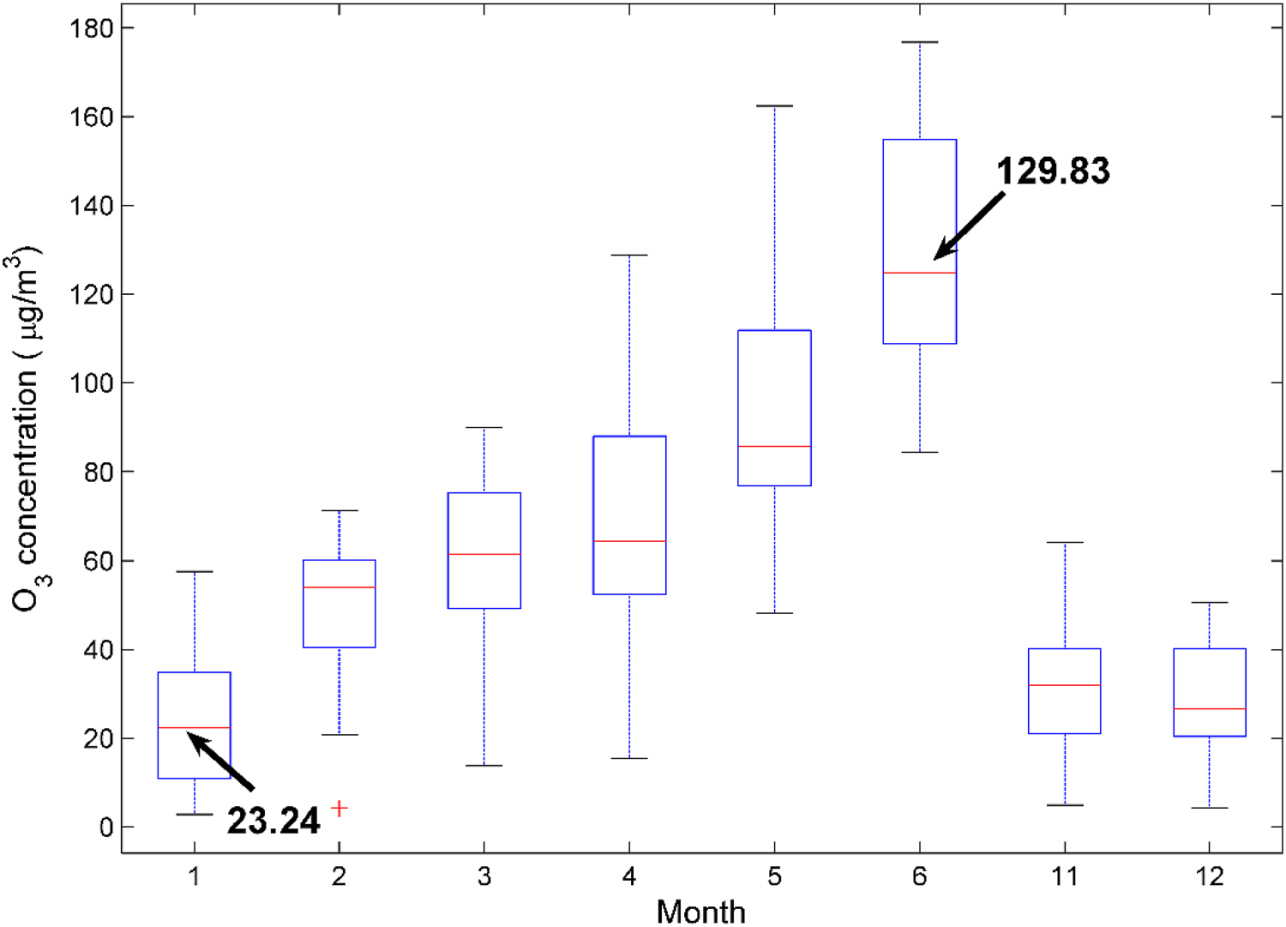
Compare the concentration of O_3_ in national control points monthly. Note that there is no data from July to October.

Figure 2 shows that the highest O_3_ concentration is in June, and the lowest O_3_ concentration is in December (no data from July to October). O_3_ pollution has obvious seasonal characteristics^32^. Near-ground ozone is mostly generated by the secondary conversion of nitrogen oxides and volatile organic compounds under high temperature and strong light conditions. The strong solar radiation and high temperature in summer can easily cause photochemical smog and secondary ozone production. Continuous high temperature and strong sunshine weather is conducive to atmospheric photochemical reaction of nitrogen oxides and volatile organic compounds, thereby generating strong oxidants such as near-ground ozone. Therefore, the O_3_ concentration in summer will increase as the temperature rises.

### Correlation analysis

Correlation mainly describes a potential relationship between two attributes. This relationship measures the degree to which one attribute contains the other. For the attribute of numerical value, the commonly used measure of correlation is the correlation coefficient. Correlation coefficients are divided into Pearson correlation coefficients, Spearman correlation coefficients and so on according to the applicable data types. The Pearson correlation coefficient measures the degree of linear correlation between two continuous numerical attributes, and the Spearman correlation coefficient mainly describes the degree of correlation between hierarchical or ordered attributes. In this paper, the Pearson correlation coefficient (Eq. 1) is selected as the evaluation index to measure the correlation between various pollutants and meteorological parameters. The absolute value of the correlation coefficient is between [0, 1]. An absolute value of 0 indicates that the two attributes are completely unrelated, and an absolute value of 1 indicates that the two attributes are completely related. The larger the absolute value of the correlation coefficient, the stronger the correlation.

**Figure 3:**
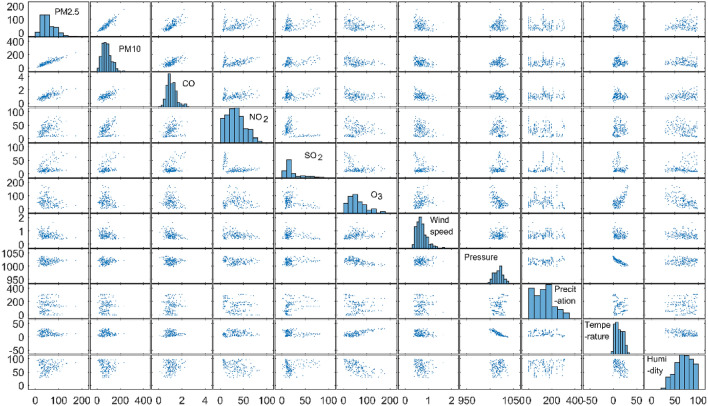
Scatter plot of the variables used for the pollutant concentration prediction model. The graph on the diagonal is the histogram of the frequency distribution of each variable.

It can be seen from Table 2 that among the 11 variables, only the NO_2_ concentration and temperature are not significantly correlated, and there is a significant correlation between the other variables. Figure 3 is a scatter plot of correlations between various variables. From the diagonal frequency histogram, it can be seen that the concentrations of the six types of pollutants all present a right-skewed distribution, indicating that extreme weather with high pollutant concentrations often occurs in this area. Most of the scatter plots between different variables are near a straight line, indicating that there is a certain linear correlation between them.

1 $$r = \frac{{\mathop \sum \nolimits_{i = 1}^{n} (x_{i} - \overline{x})\left( {y_{i} - \overline{y}} \right)}}{{\sqrt {\mathop \sum \nolimits_{i = 1}^{n} (x_{i} - \overline{x})^{2} } \cdot \sqrt {\mathop \sum \nolimits_{i = 1}^{n} (y_{i} - \overline{y})^{2} } }}$$

**Table 2:** Pearson linear correlation coefficients between six types of air pollutant concentrations and climate (Band * indicates significant correlation at a significant level of 0.05).

Variable	PM2.5	PM10	CO	NO_2_	SO_2_	O_3_	Wind speed	Pressure	Precipitation	Temperature	Humidity
PM2.5	1.00	0.89*	0.66*	0.26*	0.29*	− 0.26*	− 0.23*	0.89*	− 0.70*	− 0.16*	0.18*
PM10		1.00	0.63*	0.34*	0.35*	− 0.19*	− 0.18*	0.38*	− 0.10*	− 0.03*	− 0.09*
CO			1.00	0.30*	0.31*	− 0.27*	− 0.31*	− 0.07*	0.08*	− 0.05*	0.22*
NO_2_				1.00	− 0.34*	− 0.26*	− 0.36*	− 0.10*	− 0.14*	− 0.02	− 0.11*
SO_2_					1.00	− 0.28*	− 0.19*	0.19*	0.27*	− 0.10*	0.11*
O_3_						1.00	0.39*	− 0.45*	− 0.12*	0.68*	− 0.62*
Wind speed							1.00	0.09*	0.06*	0.07*	− 0.32*
Pressure								1.00	0.23*	− 0.85*	0.15*
Precipitation									1.00	− 0.14*	0.86*
Temperature										1.00	− 0.49*
Humidity											1.00

## Establishment of sensor calibration model

### Introduction to basic principles

The classical least square estimation has been widely used due to its many excellent properties. With the development of electronic computing technology, more and more accumulated experience in dealing with large-scale regression problems show that the results obtained by least square estimation are sometimes very unsatisfactory. When the design matrix $X$ is ill-conditioned, there is a strong linear correlation between the column vectors of $X$, that is, there is serious multicollinearity between the independent variables. In this case, using ordinary least squares to estimate the model parameters, the variance of the parameters obtained is too large, and the effect of ordinary least squares becomes very unsatisfactory.

Aiming at the problem that the ordinary least squares method obviously deteriorates when multicollinearity occurs, the American scholar Hoerl proposed an improved least squares estimation method called ridge estimation in 1962. Later Hoerl and Kennard made a systematic discussion in 1970^33^. When there is multicollinearity between the independent variables, then $\left| {X^{\prime}X} \right| \approx 0$. We add a matrix $kI(k > 0)$ to $X^{\prime}X$, then the degree to which matrix $X^{\prime}X + kI$ is close to singularity will be much smaller than the degree to which matrix $X^{\prime}X$ is close to singularity. Taking into account the dimension of variables, this article first standardizes the data. For the convenience of writing, the standardized design matrix is still denoted by $X$. Equation (2) is defined as the ridge regression estimation of $\beta$, where $k$ is called the ridge parameter. Since $X$ is assumed to have been standardized, $X^{\prime}X$ is the sample correlation matrix of the independent variables. $\hat{\beta }\left( k \right)$ as the estimate of $\beta$ is more stable than the least square estimation $\hat{\beta }$. When $k = 0$, the ridge estimation $\hat{\beta }\left( 0 \right)$ is the ordinary least square estimation. Because the ridge parameter $k$ is not unique, the ridge regression estimate $\hat{\beta }\left( k \right)$ is actually an estimated family of the regression parameter $\beta$. For the selection of the ridge parameter $k$, the commonly used methods include the ridge trace method and the variance inflation factor method.

2 $$\hat{\beta }\left( k \right) = \left( {X^{\prime}X + kI} \right)^{ - 1} X^{\prime}y$$

The XGBoost algorithm is based on an integrated learning method. The integrated learning method combines multiple learning models so that the combined model has stronger generalization ability to obtain better modeling effects. XGBoost is an improvement on the boosting algorithm based on the gradient descent tree. It is composed of multiple decision tree iterations. XGBoost first builds multiple CART (Classification and Regression Trees) models to predict the data set, and then integrates these trees as a new tree model. The model will continue to iteratively improve, and the new tree model generated in each iteration will fit the residual of the previous tree. As the number of trees increases, the complexity of the ensemble model will gradually increase until it approaches the complexity of the data itself, at which point the training achieves the best results. Equation (3) is the XGBoost algorithm model, where $f_{t} \left( {x_{i} } \right) = \omega_{q} \left( x \right)$ is the space of CART, $\omega_{q} \left( x \right)$ is the score of sample $x$, the model prediction value is obtained by accumulation, and q represents the structure of each tree , $T$ is the number of trees, and each $f_{t}$ corresponds to an independent tree structure q and leaf weight.

3 $$\hat{y}_{i} = \varphi \left( {x_{i} } \right) = \mathop \sum \limits_{t = 1}^{T} f_{t} \left( {x_{i} } \right)$$

XGBoost internal decision tree uses regression tree. For the squared loss function, the split node of the regression tree fits the residual. For the general loss function (gradient descent), the split node of the regression tree fits the approximate value of the residual. Therefore, the accuracy of XGBoost will be higher. Equations (4)–(7) are the iterative process of residual fitting. In Eq. (7), $\hat{y}_{i}^{{\left( {t - 1} \right)}}$ is the predicted value of the i-th sample after t-1 iterations. $\hat{y}_{i}^{\left( 0 \right)}$ is the initial value of the i-th sample.

4 $$\hat{y}_{i}^{\left( 0 \right)} = 0$$

5 $$\hat{y}_{i}^{\left( 1 \right)} = f_{1} \left( {x_{i} } \right) = \hat{y}_{i}^{\left( 0 \right)} + f_{1} \left( {x_{i} } \right)$$

6 $$\hat{y}_{i}^{\left( 2 \right)} = f_{1} \left( {x_{i} } \right) + f_{2} \left( {x_{i} } \right) = \hat{y}_{i}^{\left( 1 \right)} + f_{2} \left( {x_{i} } \right)$$

7 $$\hat{y}_{i}^{\left( t \right)} = \mathop \sum \limits_{k = 1}^{T} f_{k} \left( {x_{i} } \right) = \hat{y}_{i}^{{\left( {t - 1} \right)}} + f_{t} \left( {x_{i} } \right)$$

The objective optimization function of the XGBoost algorithm, that is, the loss function (Eq. 8), can be obtained according to the iterative process of the residuals. For the general loss function, XGBoost will perform a second-order Taylor expansion in order to dig out more information about the gradient, and at the same time remove the constant term, so that the gradient descent method can be better trained. Equations (9) and (10) are the loss function of the t-th step, where $g_{i}$ and $h_{i}$ are the first and second derivatives.

8 $$f_{obj}^{t} = \mathop \sum \limits_{i = 1}^{n} l\left( {y_{i} ,\hat{y}_{i}^{\left( t \right)} } \right) + \mathop \sum \limits_{i = 1}^{t} \Omega \left( {f_{i} } \right) = \hat{y}_{i}^{{\left( {t - 1} \right)}} + f_{t} \left( {x_{i} } \right) = \mathop \sum \limits_{i = 1}^{n} l\left( {y \cdot \hat{y}_{i}^{\left( t \right)} } \right) + \Omega \left( {f_{i} } \right) + C$$

9 $$g_{i} = \partial_{{\hat{y}_{i}^{{\left( {t - 1} \right)}} }} l\left( {y_{i} ,\hat{y}_{i}^{{\left( {t - 1} \right)}} } \right)$$

10 $$h_{i} = \partial_{{\hat{y}_{i}^{{\left( {t - 1} \right)}} }}^{2} l\left( {y_{i} ,\hat{y}_{i}^{{\left( {t - 1} \right)}} } \right)$$

11 $$\Omega \left( f \right) = \gamma T + \frac{1}{2}\lambda \mathop \sum \limits_{i = 1}^{n} \omega_{j}^{2}$$

Different from other algorithms, the XGBoost algorithm adds a regularization term $\Omega \left( f \right)$ (Eq. (11)) to prevent over-fitting and better improve the accuracy of the model. $\Omega \left( f \right)$ is a function that represents the complexity of the tree. The smaller the function value, the stronger the generalization ability of the tree. $\omega_{j}$ is the weight on the j-th leaf node in the tree f, $T$ is the total number of leaf nodes in the tree, ${\upgamma }$ is the penalty term of the L1 regularity, and ${\uplambda }$ is the penalty term of the L2 regularity, which is the custom parameter of the algorithm. Therefore, the objective function (Eqs. (12)–(14)) are obtained, where $I_{j} = \left\{ {\left. i \right|q\left( {x_{i} } \right) = j} \right\}$ represents the sample set on the j-th leaf node^28,34^.

12 $$f_{obj} = - \frac{1}{2}\mathop \sum \limits_{j = 1}^{T} \frac{{G_{j}^{2} }}{{H_{j} + \lambda }} + \gamma T$$

13 $$G_{j} = \mathop \sum \limits_{{i \in I_{j} }} g_{i}$$

14 $$H_{j} = \mathop \sum \limits_{{i \in I_{j} }} h_{i}$$

### Ridge regression model construction

Classical least squares estimation is often used to build pollutant concentration prediction models. It can also derive the quantitative relationship between the various influencing factors and the concentration of pollutants^15^. However, the factors that affect the concentration of pollutants are more complicated, and through the previous correlation analysis, it can be seen that there is a significant correlation between them. If the multiple linear regression model is directly established, multicollinearity will be generated, which will cause the model's regression coefficients to be very unstable, and the model application ability will deteriorate. Ridge regression is often used to solve the problem of model multicollinearity. We take the national control point O_3_ as the dependent variable, the pollutant concentration and meteorological parameters measured at the self-built point as the independent variables, and establish a ridge regression model with the help of SPSS (Version20.0,https://www.ibm.com/cn-zh/analytics/spss-statistics-software).

**Figure 4:**
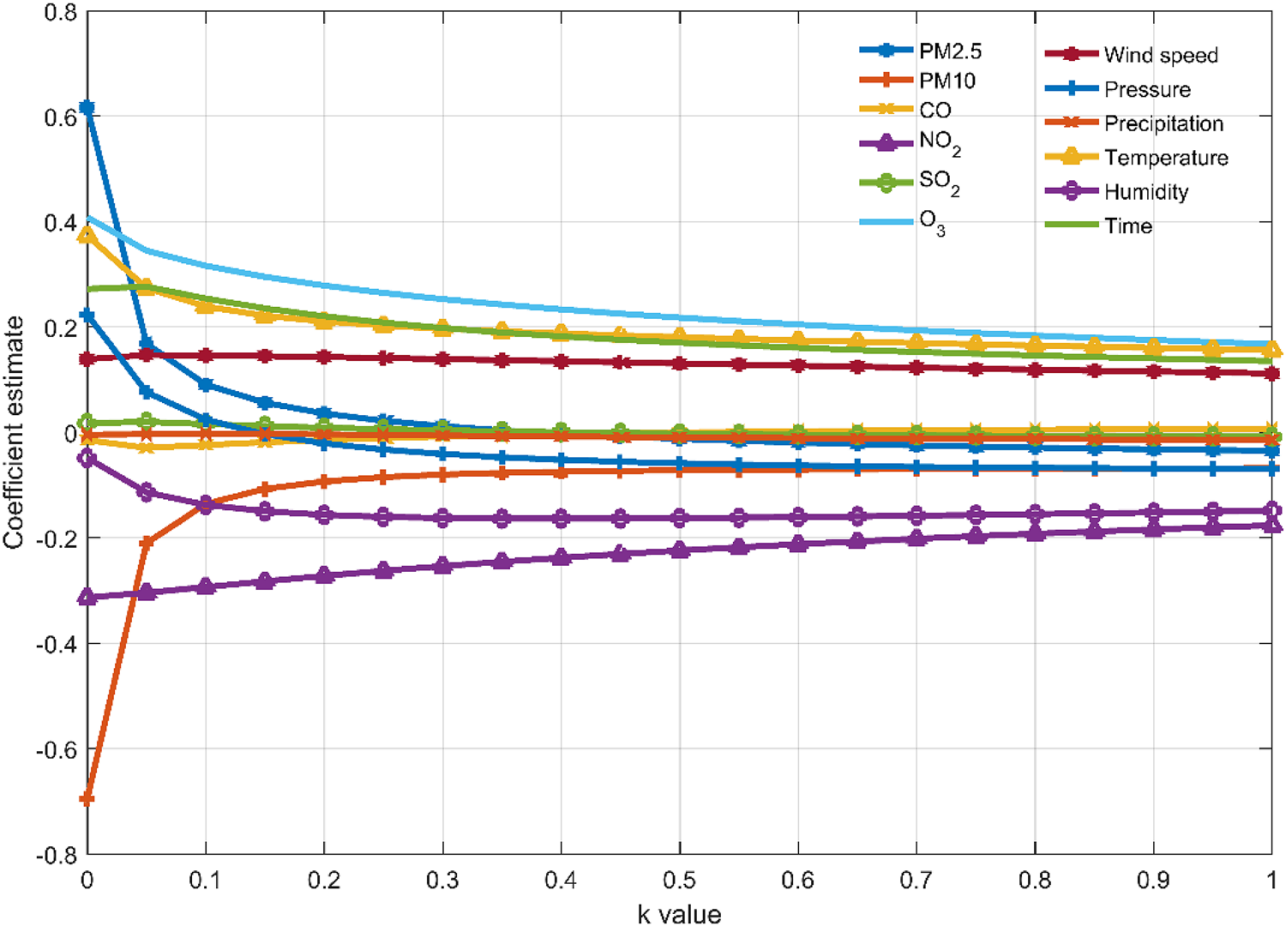
The ridge trace diagram of all input variables, where the dependent variable is the O_3_ concentration measured by the national control point.

In this paper, the ridge trace method is used to select the independent variables introduced into the model and the ridge parameter $k$. In Fig. 4, the abscissa represents the value of the ridge parameter $k$, and each curve represents the standardized ridge regression coefficient of each variable. It can be seen that $x_{4}$, $x_{6}$, and $x_{10}$ have relatively stable ridge regression coefficients with relatively small absolute values, indicating that these variables have a small impact on the O_3_ concentration, and they can be deleted in the actual modeling. In addition, although the standardized ridge regression coefficient of $x_{2}$ is not small, it is very unstable, and rapidly tends to zero as $k$ increases. For this kind of variable whose ridge regression coefficient is not stable and the rapid vibration tends to zero, it can also be eliminated in the ridge regression model.

**Figure 5:**
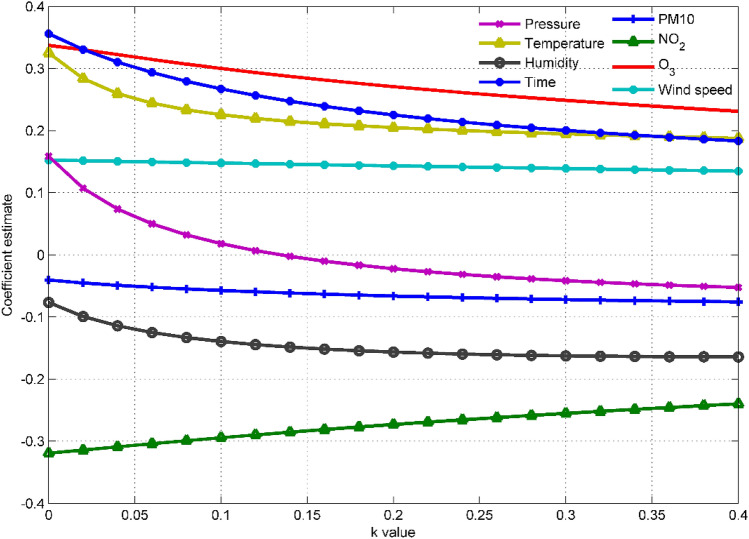
The ridge trace diagram of the input variable after the variable selection is completed, where the dependent variable is the O_3_ concentration measured by the national control point.

After completing the selection of the independent variables of the ridge regression model, the next step is the selection of the ridge parameter $k$. We reduce the step length of the ridge parameter $k$ to 0.02, and draw the ridge trace diagram of the remaining variables as Fig. 5. It can be seen that when the ridge parameter $k = 0.2$, the ridge trace of each variable is relatively stable, and the coefficient of determination *R*^2^ is not reduced much, so the ridge parameter $k = 0.2$ can be selected. Finally, with the help of SPSS software, use the selected variables and ridge parameters to make a ridge regression model. Table 3 shows the unstandardized ridge regression equations for six types of pollutants. Using these equations, the predicted value of the ridge regression model for the concentration of each pollutant can be obtained.

**Table 3:** Ridge regression model of six types of air pollutant concentrations.

Independent variable	PM2.5	PM10	CO ($\times 10^{ - 2}$)	NO_2_	SO_2_	O_3_
Constant	359.559	374.836	684.980	40.691	13.826	160.997
PM2.5	0.48	0.493	0. 375	0.089	–	–
PM10	0.162	0.203	0. 141	–	0.022	− 0.043
CO	4.713	21.544	26.741	− 6.764	20.205	–
NO_2_	0.083	0.307	0. 26	0.298	0.049	− 0.494
SO_2_	–	0.121	–	–	–	–
O_3_	–	–	0.038	− 0.09	0.082	0.418
Wind speed	− 1.107	–	− 11.773	− 12.601	− 6.101	19.890
Pressure	− 0.336	− 0.319	− 0. 638	–	–	− 0.121
Precipitation	− 0.036	− 0.073	–	− 0.028	0.020	–
Temperature	–	–	–	− 0.316	0.219	1.143
Humidity	− 0.257	− 0.747	0.011	− 0.237	− 0.034	− 0.342
Time	–	–	0.003	0.005	− 0.007	0.009
k value	0.12	0.18	22	0.38	0.26	0.2
*R*^2^	0.896	0.787	48.326	0.497	0.517	0.787

In the model, the dependent variable is the concentration of the six pollutants at the national control point, and the independent variable is the variable and time monitored by the self-built point (– represents the variables eliminated in the model).

### RR-XGBoost model construction

**Figure 6:**
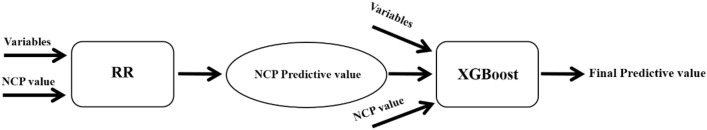
The flux diagram of the regression process, where NCP represents the concentration of pollutants measured at the national control point.

The ridge regression model can be used to predict the concentration of pollutants, and it can also show the quantitative relationship of the influence of each input variable on the concentration of pollutants. However, ridge regression can only show the linear relationship between variables, while the nonlinear relationship between various factors and pollutant concentration has not been found. This study uses the ridge regression prediction value and self-built point measurement data as input, and uses the pollutant concentration value monitored by the national control point as the output. The XGBoost algorithm is used to establish a prediction model for the concentration of each pollutant. We call this model the RR-XGBoost model. Figure 6 is the flux diagram of the RR-XGBoost model.

Before constructing the Ridge-XGBoost model, first divide all samples into training set and test set randomly at a ratio of 8:2 (the other 5 pollutants data sets are also divided in the same way), and normalize all data to the range of [0,1] based on experience^29,34^. The modeling in this paper is implemented using Python language programming, the simulation platform is Pycharm, and the Grid Search Method (GSM) is used to find the optimal parameter combination.

The XGBoost model has many parameters. If all parameters are optimized, the computer's memory will be challenged and the optimization time will be greatly increased. In this paper, the following four main parameters are selected for optimization: (i) the number of gradient boosted trees n_estimators, the larger the parameter, the better, but the occupied memory and training time will also increase accordingly, the optimization range of this article is 100–300; (ii) the maximum tree depth for base learners max_depth, this parameter is used to avoid overfitting, the value range is 3–10; (iii) learning rate learning_rate, the value range is 0.01–0.3; and (iv) the minimum sum of instance weight(hessian) needed in a child min_child_weight, which is similar to max_depth, used to avoid over-fitting, and the value range is 1–9. The four initial parameters of the XGBoost model are set to 100, 6, 0.1, and 1. In addition, GSM needs to set the optimization step distance of each parameter during the optimization process (this article takes 10, 1, 0.01, 1).

**Figure 7:**
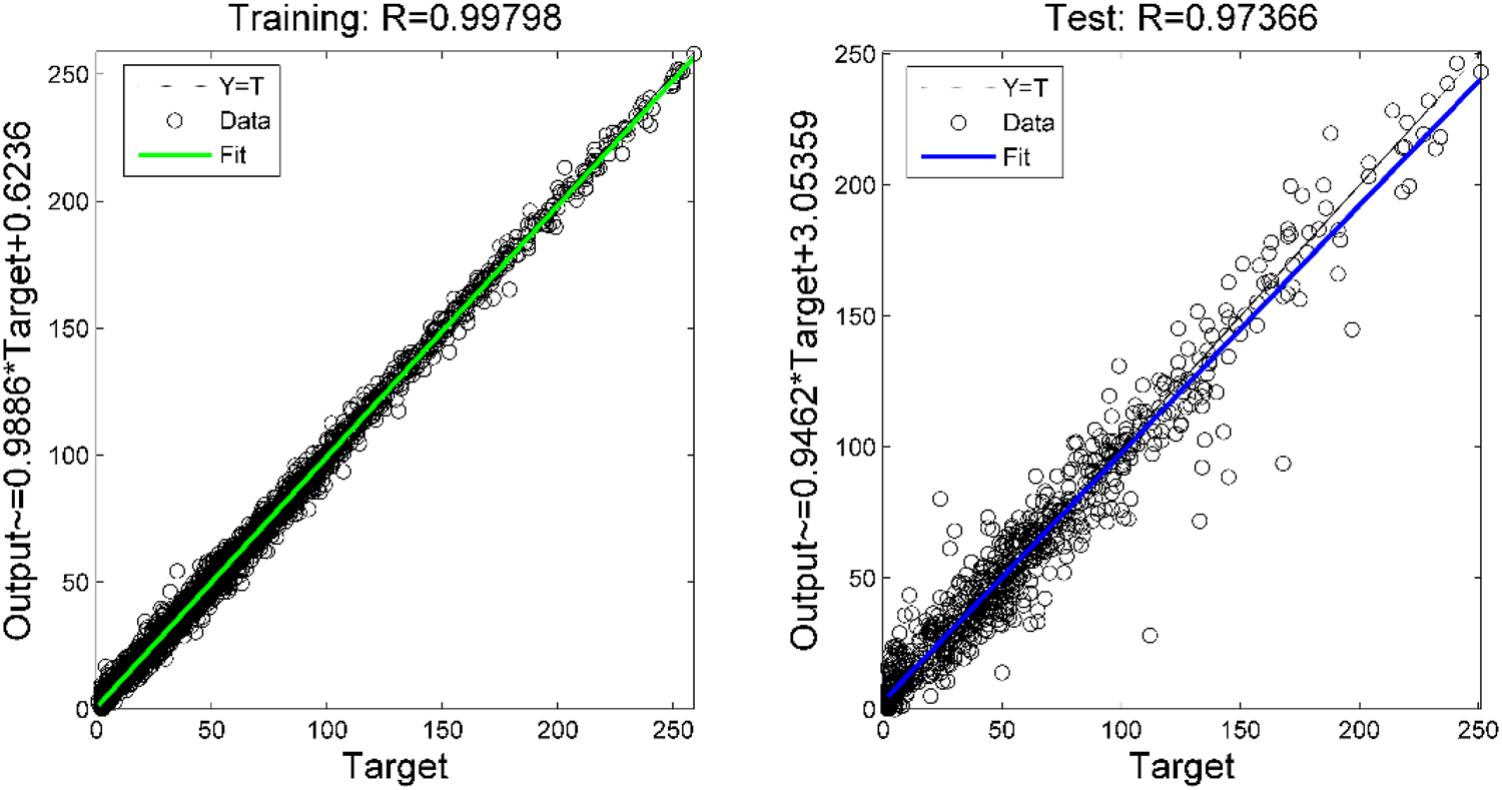
The prediction effect of O_3_'s RR-XGBoost model on the training set and test set.

**Figure 8:**
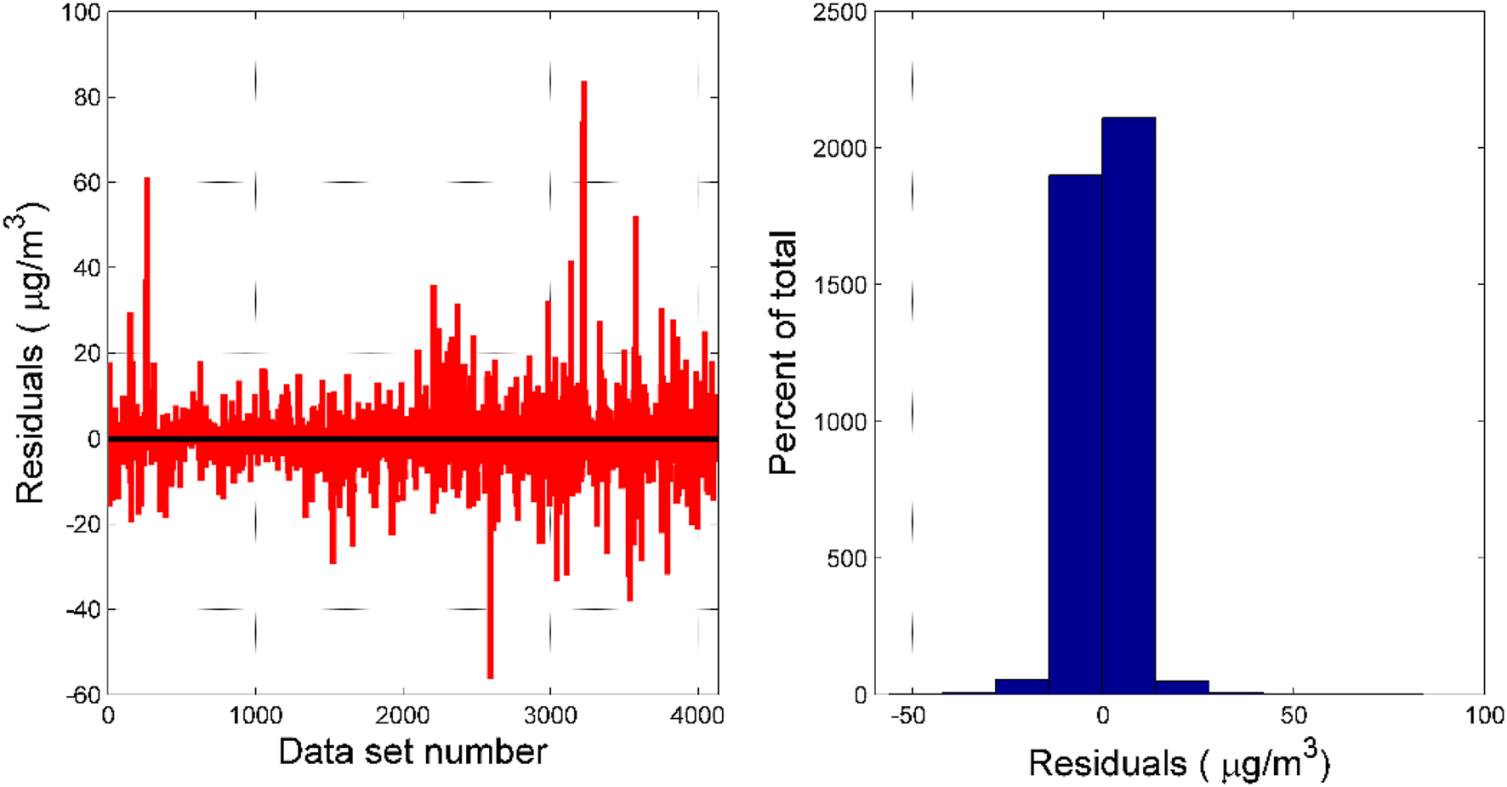
Residual test of RR-XGBoost model. The residuals vs. data set number plot is seen on the left. The histogram of the residuals is seen on the right.

Table 4 shows the parameters of the XGBoost model determined after using the grid search method. In order to show the fitting effect of the RR-XGBoost model more intuitively, this paper draws the fitting effect of O_3_ concentration as shown in Fig. 7. It can be seen that the correlation coefficient between the true concentration of O_3_ and the predicted concentration of the model in both the training set and the test set exceeds 0.95. In addition, the regression coefficients of the two regression models (training set regression model and test set regression model) are close to 1, indicating that this model performs well in predicting the concentration of pollutants. Figure 8 is the residual analysis diagram of the RR-XGBoost model. It can be seen that most of the residual values of the model are randomly distributed within [-40, 40]. From the residual distribution histogram, it can be seen that the residuals are uniformly distributed around zero, and the residuals are roughly normally distributed as a whole.

**Table 4:** Six types of pollutant concentration prediction model parameters.

Model parameters	PM2.5	PM10	CO	NO_2_	SO_2_	O_3_
n_estimators	210	290	290	200	300	300
max_depth	6	7	7	7	10	6
learning_rate	0.10	0.08	0.10	0.06	0.10	0.10
min_child_weight	1	7	1	3	1	7

## Discussion

In order to further evaluate the prediction accuracy of the RR-XGBoost model, multilayer perceptron neural network, random forest regression and support vector machine were used to compare with this model. This study uses four commonly used evaluation indicators to compare each model. The four evaluation indicators are relative Mean Absolute Percent Error (MAPE), Mean Absolute Error (MAE), goodness of fit (*R*^2^), and Root Mean Square Error (RMSE) (Eqs. (15)–(18)). From Tables 5, 6, 7, and 8, it can be seen that the measurement accuracy of self-built points is the lowest among all evaluation indicators, which shows that the measurement accuracy of the micro air quality detector needs to be improved. Although ridge regression can give the quantitative relationship between each variable and the concentration of pollutants, the fitting effect is not particularly good. Random forest regression and XGBoost prediction methods are better in the accuracy of pollutant concentration prediction. In particular, the XGBoost prediction method can greatly improve the accuracy of pollutant concentration prediction. The model combining ridge regression and XGBoost algorithm presented in this study is not only slightly higher in accuracy than the single XGBoost prediction method, but also retains the advantages of ridge regression model.

15 $$MAPE = \frac{1}{n}\mathop \sum \limits_{t = 1}^{n} \left| {\frac{{y_{t} - w_{t} }}{{y_{t} }}} \right|$$

16 $$MAE = \frac{1}{n}\mathop \sum \limits_{t = 1}^{n} \left| {y_{t} - w_{t} } \right|$$

17 $$R^{2} = 1 - \frac{{\mathop \sum \nolimits_{t = 1}^{n} \left( {y_{t} - w_{t} } \right)^{2} }}{{\mathop \sum \nolimits_{t = 1}^{n} \left( {y_{t} - \overline{y}} \right)^{2} }}$$

18 $$RMSE = \sqrt {\frac{1}{n}\mathop \sum \limits_{t = 1}^{n} \left( {y_{t} - w_{t} } \right)^{2} }$$

**Table 5:** MAPE of six types of air pollutant concentrations between self-built points, model forecast values and national control point.

Input variable	Self-built points	Ridge	XGBoost	RR-XGBoost	RFR	SVR	MLP
PM2.5	0.447	0.186	0.067	0.064	0.087	0.133	0.185
PM10	0.887	0.268	0.061	0.055	0.095	0.107	0.210
CO	0.478	0.332	0.038	0.037	0.083	0.112	0.283
NO_2_	2.129	0.659	0.092	0.088	0.121	0.170	0.471
SO_2_	0.685	0.645	0.029	0.029	0.115	0.131	0.530
O_3_	4.322	1.259	0.177	0.167	0.304	0.373	1.002

**Table 6:** MAE of six types of air pollutant concentrations between self-built points, model forecast values and national control point.

Input variable	Self-built points	Ridge	XGBoost	RR-XGBoost	RFR	SVR	MLP
PM2.5	18.181	7.634	2.552	2.491	3.485	5.821	7.763
PM10	50.151	15.027	3.870	3.477	6.299	7.080	13.184
CO	0.549	0.269	0.036	0.035	0.079	0.110	0.237
NO_2_	29.838	13.078	2.570	2.441	3.515	4.658	9.991
SO_2_	12.867	9.299	0.555	0.538	1.736	2.116	7.246
O_3_	36.63	17.239	3.536	3.267	5.638	7.647	14.396

**Table 7:** *R*^2^ of six types of air pollutant concentrations between self-built points, model forecast values and national control point.

Input variable	Self-built points	Ridge	XGBoost	RR-XGBoost	RFR	SVR	MLP
PM2.5	0.551	0.8959	0.986	0.986	0.976	0.933	0.907
PM10	− 1.076	0.7867	0.985	0.987	0.953	0.938	0.827
CO	− 0.929	0.4833	1.000	1.000	0.932	0.872	0.708
NO_2_	− 1.333	0.4967	0.982	0.982	0.942	0.899	0.752
SO_2_	− 0.726	0.5168	0.997	0.997	0.969	0.958	0.786
O_3_	0.094	0.7866	0.985	0.986	0.969	0.945	0.864

**Table 8:** RMSE of six types of air pollutant concentrations between self− built points, model forecast values and national control point.

Input variable	Self-built points	Ridge	XGBoost	RR-XGBoost	RFR	SVR	MLP
PM2.5	22.436	10.802	3.992	3.976	5.207	8.649	10.777
PM10	66.263	21.232	6.479	6.032	9.940	11.656	19.126
CO	0.679	0.352	0.079	0.078	0.128	0.175	0.304
NO_2_	37.183	17.271	4.502	4.507	5.847	7.725	13.216
SO_2_	26.24	13.882	2.236	2.163	3.513	4.116	9.984
O_3_	45.673	22.169	5.798	5.669	8.433	11.304	18.603

**Figure 9:**
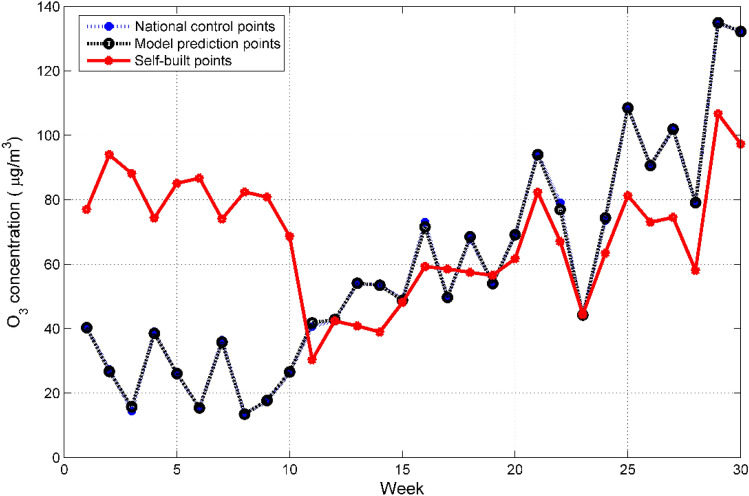
Comparison of the weekly average concentration of O_3_ between national control points, RR-XGBoost model calibration points and self-built points.

Human activities are one of the important factors affecting the concentration of pollutants. Human activities have obvious periodic laws. We choose one week as a cycle to evaluate the correction ability of the RR-XGBoost model to the measurement data of the micro air quality detector^35^. The blue curve in Fig. 9 is the measured value of the national control point, the red curve is the measured value of the self-built point, and the black curve is the predicted value of the RR-XGBoost model. It can be seen that the red curve and the blue curve have a certain error, but the black curve and the blue curve basically overlap, indicating that the RR-XGBoost model has performed a good correction on the measurement data of the micro air quality detector.

## Conclusions

Today, the situation of air pollution is still not very optimistic^3^, and atmospheric monitoring is gradually developing in the direction of refined monitoring. At present, the most feasible solution for refined atmospheric monitoring is grid-based monitoring, that is, multiple air quality monitoring devices are set up within a certain distance or range in a monitoring area to measure the specific dust particle concentration and pollutant gas concentration. A city will set up dozens to hundreds of monitoring points. Accurate and fine grid air monitoring can quickly perceive and locate pollution events, and timely take control measures to achieve a multiplier control and governance effect^5,7^. At present, many places use such micro-stations for the detection and law enforcement of sudden pollution situations, and even rank, reward and punish the air quality in the jurisdiction. Therefore, higher requirements are put forward for the stability and accuracy of the micro air quality inspection station.

With the development of computer technology, machine learning has entered the latest stage, and machine learning has been more widely used in air quality prediction. The XGBoost algorithm is widely used in data modeling due to its excellent computational efficiency and prediction accuracy. Unlike the random forest assigning the same voting weight to each decision tree, the generation of the next decision tree in the XGBoost algorithm is related to the training and prediction of the previous decision tree. The XGBoost algorithm gives higher learning weights to the sample which has lower accuracy in the previous round of decision tree training. Therefore, its accuracy is generally higher than the random forest algorithm. Compared with other ensemble learning algorithms, XGBoost improves the robustness of the model by introducing regular terms and column sampling methods. On the other hand, it adopts a parallelization strategy when each tree chooses the split point, which greatly improves the speed of the model.

The combined model of ridge regression and XGBoost algorithm given in this paper can not only explain the quantitative relationship between input variables and output variables, but also has certain advantages over other commonly used air quality monitoring models in terms of model accuracy. A total of 4135 samples were introduced into the Ridge-XGBoost model, and the sample time spanned 4 seasons (206 days), which showed that the model performed well in terms of stability. Using the RR-XGBoost model to calibrate the data of the micro air quality detector can make up for the shortcomings of the data monitoring accuracy of the micro air quality detector. The model plays an active role in the deployment of micro air quality detectors and grid monitoring of the atmosphere. In future research, we can consider introducing more data to explore the evolution of pollutant concentrations on a larger time scale. In addition, in terms of finding the optimal parameters, the grid search algorithm used in this study is not efficient enough when there are many parameters. We can try to find a more efficient parameter optimization method to introduce more parameters to the model to further improve the accuracy of the model.
